# Peripherally inserted central catheters versus implantable port catheters for cancer patients: a meta-analysis

**DOI:** 10.3389/fonc.2023.1228092

**Published:** 2023-07-14

**Authors:** Li Lin, Wei Li, Chen Chen, Anhua Wei, Yu Liu

**Affiliations:** ^1^ Department of Oncology, Wuhan Asia General Hospital, Wuhan, China; ^2^ Department of Pharmacy, Tongji Hospital, Tongji Medical College, Huazhong University of Science and Technology, Wuhan, China

**Keywords:** PICC, PORT, cancer, central venous access, meta-analysis

## Abstract

**Background:**

The implanted vascular access ports (PORTs) were compared with peripherally inserted central catheters (PICCs) as the administration of chemotherapy regarding different clinical effects and adverse effects. Which is better is debatable. Hence, the current study was conducted to assess the safety and efficacy of these two optimal vascular access strategies.

**Methods:**

The following electronic databases were searched: PubMed, Embase, and the Cochrane Library updated in May 2023. Studies on the differences in complication rates in patients with cancer using either PICC or PORT for chemotherapy were included. Meta-analysis Revman 5.3 software was used for statistical analysis.

**Results:**

A total of 22 articles were retrieved. The results suggested that PORT has a superior safety profile, with lower incidences of overall adverse effects (OR=2.72, 95% CI=1.56–4.72 P=0.0004), catheter-related thrombosis (OR=2.84, 95% CI=1.97–4.11, P<0.00001), and allergic reactions (OR=6.26, 95% CI=1.86–21.09, P=0.003) than typically expected with PICC. Moreover, PICC was non-inferior to the PORT group with respect to DVT (OR=2.00, 95% CI=0.86–4.65, P=0.11) and infection (OR=1.55, 95% CI=0.75–3.22, P=0.24).

**Conclusion:**

PORT achieved safety benefits compared with chemotherapy through PICC. Therefore, PORT is regarded as safe and effective vascular access for the administration of chemotherapy. When considering economic factors and some key elements, more high-quality research would help verify these clinical benefits.

**Systematic review registration:**

https://www.crd.york.ac.uk/prospero/, identififier CRD42023421690.

## 1 Introduction

A central venous device provides safe and reliable access strategy for the central vein and avoids venous toxicities and peripheral venous punctures for chemotherapy delivery.

To date, peripherally inserted central catheters (PICCs) and implanted vascular access ports (PORTs) are two common infusion pathways representing vital developments in nursing technologies for patients receiving chemotherapy (1).

The PICC was replaced with the central venous catheter (CVC) in the 1970s and has been widely used in modern oncology departments (2). Compared with CVC, PICC has been reported to have many advantages, such as avoiding mechanical complications related to CVC, and PICC trained nurse teams have made it quicker and easier to manage in oncology practice (3). A port catheter (PC), the classical route for receiving chemotherapy, was developed to provide deep venous access and allows for iterative perfusion for years.

Recently, concerns have been raised regarding whether PICC or PORT is the optimal vascular access strategy for patients with cancer. All PICCs can be easily implanted and removed but require weekly maintenance, whereas PORTs need monthly maintenance, usually conducted at the end of the chemotherapy infusion. Many oncologists prefer PICCs over PORTs, with the advantages of ease of implantation by nursing teams without a surgical procedure and lower costs (4). Additionally, the device can be easily removed for source control in cases of infection or thrombosis. Nevertheless, previous studies have indicated a higher rate of complications among oncological patients with PICC than those with PORT (5–7).

A meta-analysis by Wang (8) found that PORTs showed favorable influence in reducing the risk of VTE than PICCs in oncologic patients. Meanwhile, some meta-analyses have been carried out to evaluate the safety efficacy of PICCs versus PORTs. However, the pool results had limitations. The research by Wang et al. (8) only explored the incidence of PORT-and PICC-related VTEs in patients with cancer. Baiying Liu’s study (9) only included patients with non-hematological malignancies. The conclusion of another meta-analysis (10) was applicable only to gynecological cancers.

Thus, a systematic, up-to-date assessment with additional trials is needed. We conducted this meta-analysis to identify evidence for differences in the complication rates of PICC versus PORT in patients with cancer who are receiving chemotherapy, including patients with hematological and non-hematological malignancies.

## 2 Materials and methods

### 2.1 Search strategy

Two reviewers separately performed literature searches of the PubMed, Embase, and Cochrane databases until May 2023. The literature searching process was conducted with the keywords: “PICC” AND “PORT” AND “cancer” AND “chemotherapy.” Selected Medical Subject Headings were combined with free text terms following Medical Subject Headings (MeSH) terms relating to PICC (peripherally inserted central catheter line insertion, peripheral catheterization, PICC Line Catheterizations), PORT (vascular access device, TIVAD, implantable access port, implantable port catheter, central venous port access system), cancer (tumor, lymphoma, carcinoma, neoplasms, sarcoma), and chemotherapy (chemical, chemo, chemotherapeutic, pharmacotherapies) in PubMed. No limitation was set during the literature search. The references of the eligible studies were checked for additional studies found in the literature search.

### 2.2 Eligibility criteria

Inclusion criteria were studies relating to the following: (1) patients: studies that enrolled patients with cancer treated with chemotherapy through central venous access devices; (2) design: articles that focused on comparing PICC and PORT; (3) outcomes of interest including the rate of adverse effects, catheter-related thrombosis, deep vein thrombosis, allergic reaction, and infection; and (4) only English texts.

Articles with the following exclusion criteria were eliminated: (1) trials without a comparison group, (2) incompletely reported data that were unable to provide research outcomes, (3) case reports or observational studies, and (4) duplicate previous literature.

### 2.3 Quality assessment

The risk of bias for each included study was evaluated based on the risk of bias items (ROBI) recommended in The Cochrane Handbook for Systematic Reviews of Interventions. All cohort studies were justified using the Newcastle–Ottawa Scale (11). The process was conducted by two researchers separately, and differences were resolved through discussion. The Newcastle–Ottawa Scale method uses three domains to assess the quality of cohort studies, which include the selection of patients with cancer, comparability between two groups, and assessment of outcomes. Based on the NOS method, four, two, and three points were awarded to the three domains, respectively. Studies with no less than seven points were identified as having high quality; however, those with six points or less were identified as having low quality.

In addition, the Cochrane Collaboration’s “Risk of bias tool for RCT was used (12). The tools for RCT scored a research article according to the descriptions of randomization (two points), blinding (two points), and attrition information (one point). A score of ≥3 points suggests that the study was of “high quality,” and a score of ≤2 points suggests that the study was of “low quality.” To minimize heterogeneity among studies, only high-quality papers were included. The included studies had comparable baseline clinical characteristics without statistically significant differences between the two groups.

### 2.4 Data extraction

Two reviewers independently extracted information from each article. Disagreements were resolved through discussion. From each of the eligible studies, the following information was collected: author’s name, year of publication, tumor type, type of PORT, date of collection, number of patients, mean age of the patients, and outcome data of interest.

### 2.5 Data synthesis and analysis

I^2^ and chi-square tests were used to assess the heterogeneity of the studies (13). Studies with I^2^≥50% were considered as high degree of heterogeneity, and I^2^<50% suggested low heterogeneity (14). A fixed-effects model was used when heterogeneity among the studies was of low degree. A random-effects model was used when the degree of heterogeneity was uncertain (I^2^>50%). Statistical significance was set at P<0.05. significant. Review Manager version 5.3 software (Revman; The Cochrane Collaboration, Oxford, United Kingdom) was used for statistical analysis. Our results are shown in forest plots, and publication bias was evaluated using funnel plots.

## 3 Results

### 3.1 Study selection

**Figure 1:**
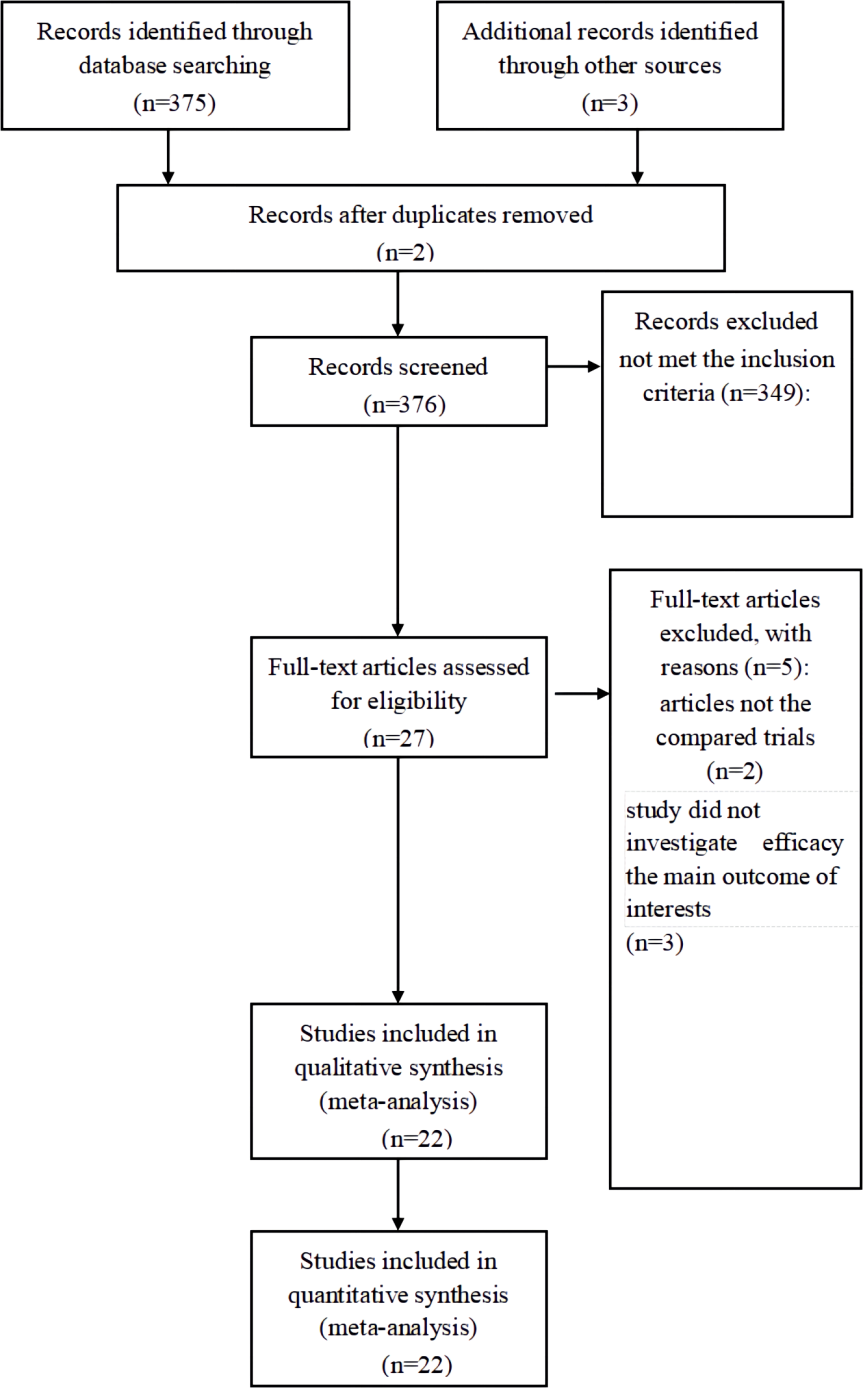
PRISMA flow chart of selection process to identify studies eligible for pooling.

**Figure 2:**
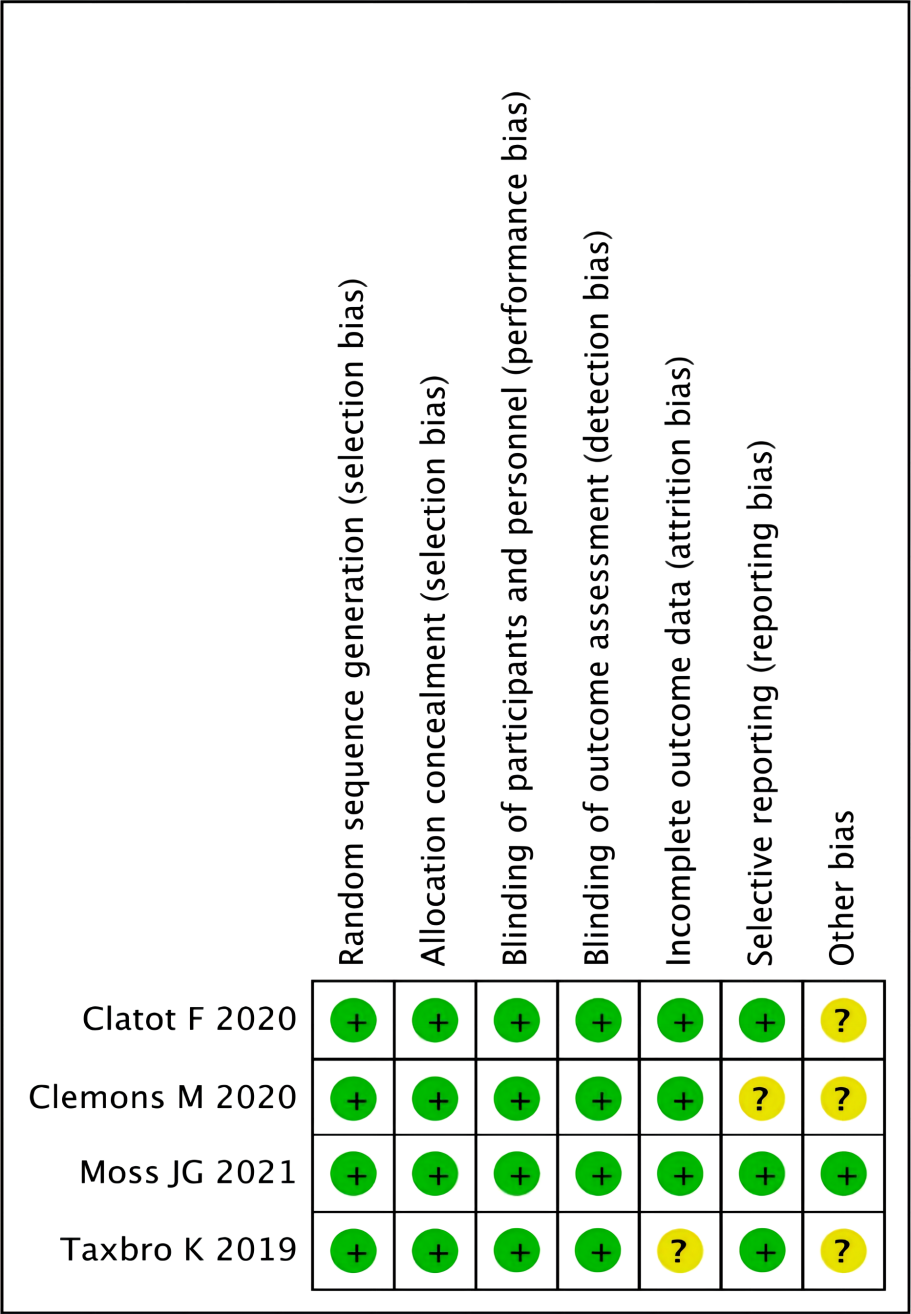
Methodological quality assessment for each RCT study.

A total of 376 publications were retrieved. During the review of abstracts and titles, 27 studies were evaluated through reading the full articles; however, five were excluded based on the inclusion criteria. Finally, 22 studies were further eliminated (15–36). **Figure 1** illustrates the search process in detail. **Figure 2** presented the summary of the quality assessment process of the RCTs. **Table 1** describes the primary characteristics of the eligible studies in detail.

**Table 1:** The primary characteristics of the eligible studies in more detail.

	Study design	Tumour type	Type of port	Date Collection Time	No. of patients		Median age		NOS
					PICC	PORT	PICC	PORT	
Martella F 2015 (15)	RS	sarcoma,ovarian cancer,cervical cancer	PORT	2009.11-2013.3	45	57	56	53	7
Yun WS 2021 (22)	RS	early-stage breast cancer	TIVADs	2016.5-2019.4	185	282	63.7	57.4	8
Tang TT 2019 (23)	RS	breast Cancer	TIVADs	2014.4-2018.3	1433	4080	43.4	50	7
Lefebvre L 2016 (17)	RS	early breast cancer	PORT	2010.1-2012.8	290	158	NA	NA	7
Patel GS 2014 (19)	RS	colorectal cancer,Upper gastrointestinal tract cancer,breast cancer,other	PORT	2004.12-2010.1	36	34	59	60	8
Fang S 2017 (20)	RS	breast cancer,lung cancer,gastrointestinal cancer,others	PORT	2014.3-2016.12	45	60	52.2	52.38	7
Yin L 2020 (31)	RS	colorectal cancer patients	PORT	2017.1.2 -2019.1.1	65	698	NA	NA	7
Akhtar N 2021 (27)	RS	breast cancer,gastrointestinal,lung cancer,gynecological cancer,haematological malignancy,skin cancer,other	PORT	2018.1.1-2018.12.31	408	72	NA	NA	7
Zhang H 2022 (28)	RS	leukemia, lymphoma, others	TIVAPs	2020.5-2021.5	48	48	78	47	8
Corti F 2021 (29)	RS	non-Hodgkin lymphoma,sarcoma,hodgkin lymphoma,breast cancer,head and neck cancer,gastrointestinal cancers,bilio-pancreatic cancers,T-cell lymphoma,non-small cell lung cancer,anal cancer,chronic lymphocytic leukemia,multiple myeloma,germinal cancer;urothelial cancer,small cell lung cancer,acute myeloid leukemia,melanoma,neuroendocrine tumor	implantable central venous catheters	2016.1-2018.12	130	48	NA	NA	7
Haggstrom L 2020 (30)	RS	breast cancer, pancreatic cancer, colorectal cancer, gastric cancer, acute lymphocytic leukae mia (ALL), acute myeloid leukaemia (AML)	PORT	2018.6-2019.3	165	236	NA	NA	7
Cotogni P 2021 (32)	RS	gastrointestinal,ovary carcinoma,others	PORT	2008.6.1-2015.5.31	401	198	NA	NA	7
Burbridge B 2021 (33)	RS	breast cancer,colon cancer	PORT	2016.1-2018.12	50	51	55.4	55.4	8
Kim H 2021 (34)	RS	breast cancer	PORT	2016.1-2018.6	100	200	51	51	8
Wu O 2021 (35)	RS	colorectal cancer,breast cancer,pancreatic cancer,others	PORT	2013.11.8-2018.2.28	199	147	61	61	8
Verboom MC 2017 (36)	RS	leiomyosarcoma,liposarcoma,synovial sarcoma,others	Venous access port	1999.11-2014.11	10	102	NA	NA	7
Wang K 2022 (25)	PSM	breast cancer,gastrointestinal,lymphoma,gynecological cancer,other	PORT	2016.1-2019.10	138	138	NA	NA	7
Shao G 2022 (26)	PSM	gastrointestinal,lung cancer,gynecological cancer,breast cancer,nasopharyngeal carcinoma,other	PORT	2021.4.6-2021.5.6	202	202	57.61	57.45	8
Clatot F 2020 (21)	RCT	early breast cancer	PORT	2014.2 -2018.5	128	128	57.5	56	/
Clemons M 2020 (18)	RCT	early-stage breast cancer	PORT	2016.3-2018.3	29	27	52	54	/
Moss JG 2021 (24)	RCT	solid or haematological malignancy	PORT	2013.11.8-2018.2.28	199	147	61	61	/
Taxbro K 2019 (16)	RCT	breast cancer,colorectal cancer,upper gastrointestinal tract cancer,urogenital cancer,other	PORT	2013.3.13-2017.2.16	201	198	66	65	/

RCT, Randomized controlled trials; RS, Retrospective study; NOS, Newcastle-Ottawa Quality Assessment Scale; NA, Not available.

The number of participants with PICC recruited varied among the 22 studies, ranging from 10 to 1433. In addition, the number of participants recruited with PORT ranged from 27 to 4080.

The quality of the included studies was assessed using the NOS method; 18 studies scored at least seven points and thus were of high quality. All the included publications were based on moderate-quality evidence (**Table 1**).

### 3.2 Clinical and methodological heterogeneity

#### 3.2.1 Overall adverse effects

**Figure 3:**
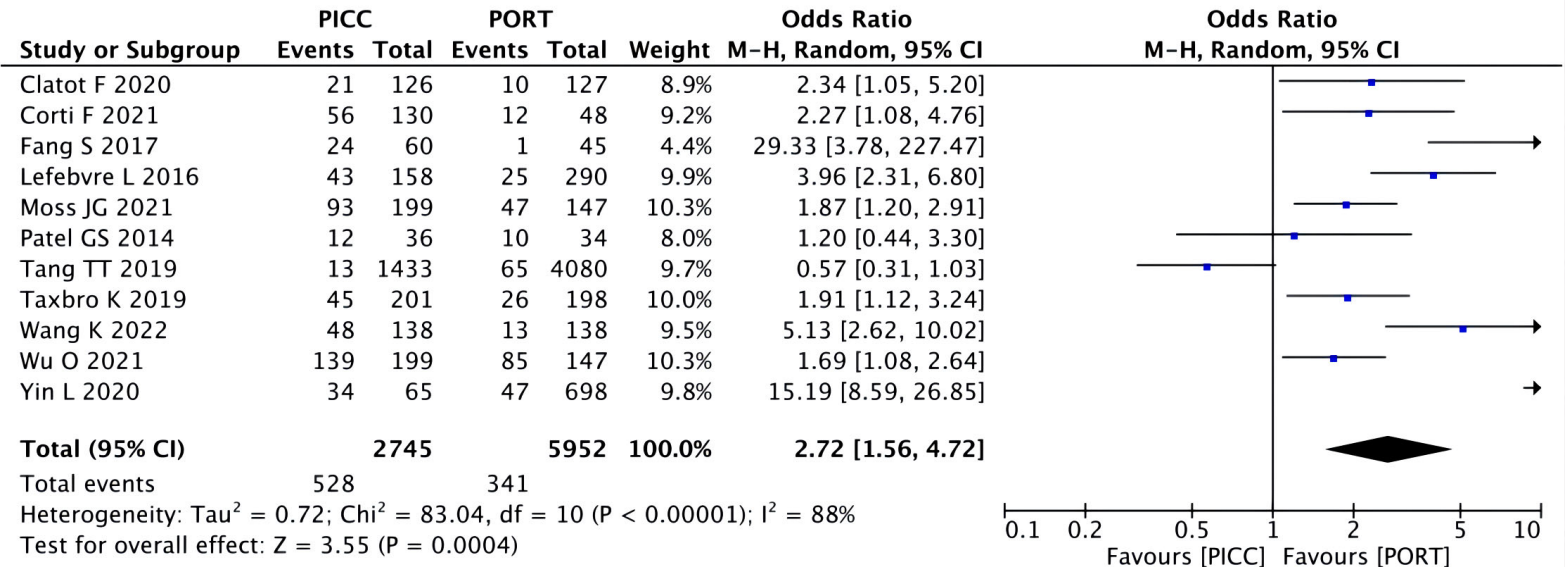
Pooled analysis of overall adverse effects comparing PICC with PORT.

Heterogeneity among the 11 studies was high (I^2 ^= 88%, P<0.00001). For the AEs, a significant difference was observed between PICC and PORT with the random-effects model (OR=2.72, 95% CI=1.56–4.72 P=0.0004) (**Figure 3**).

#### 3.2.2 Complications by access type

##### 3.2.2.1 Catheter-related and deep vein thrombosis

**Figure 4:**
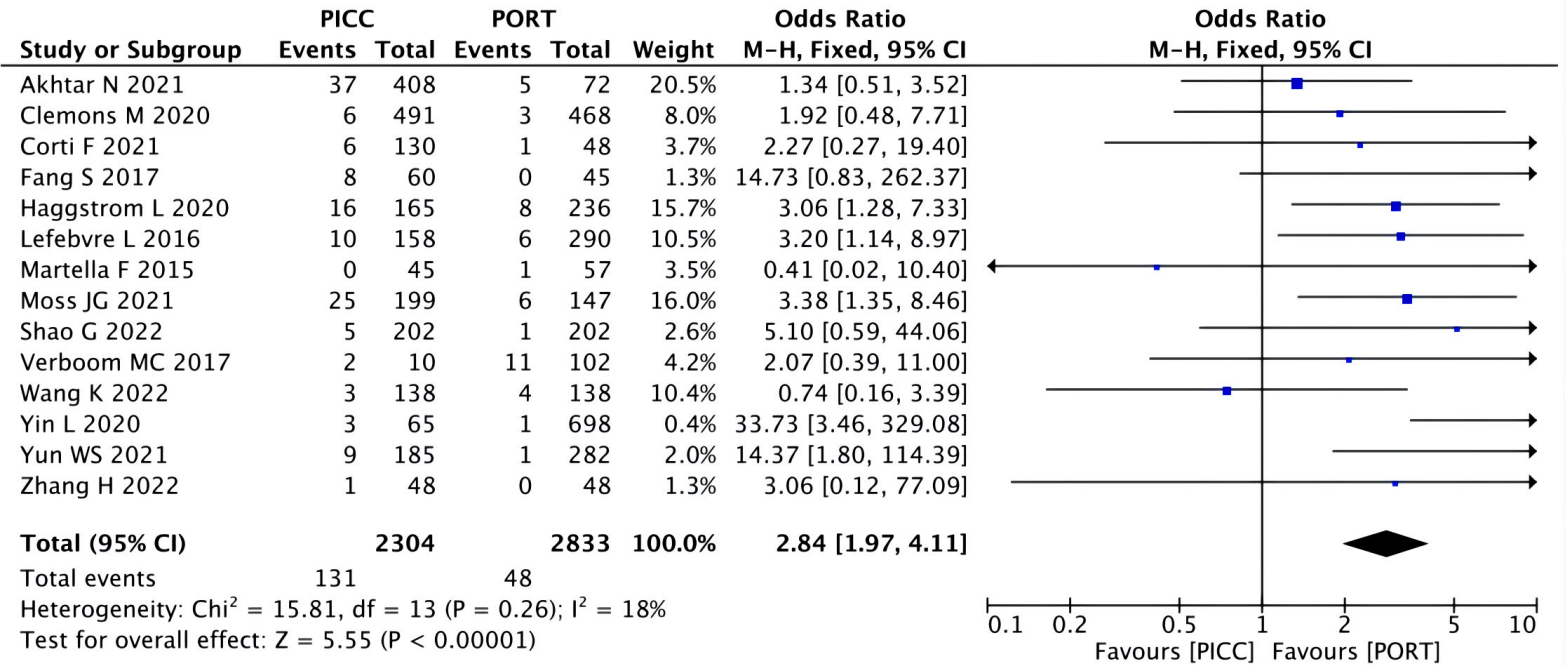
Pooled analysis of catheter-related thrombosis comparing PICC with PORT.

**Figure 5:**
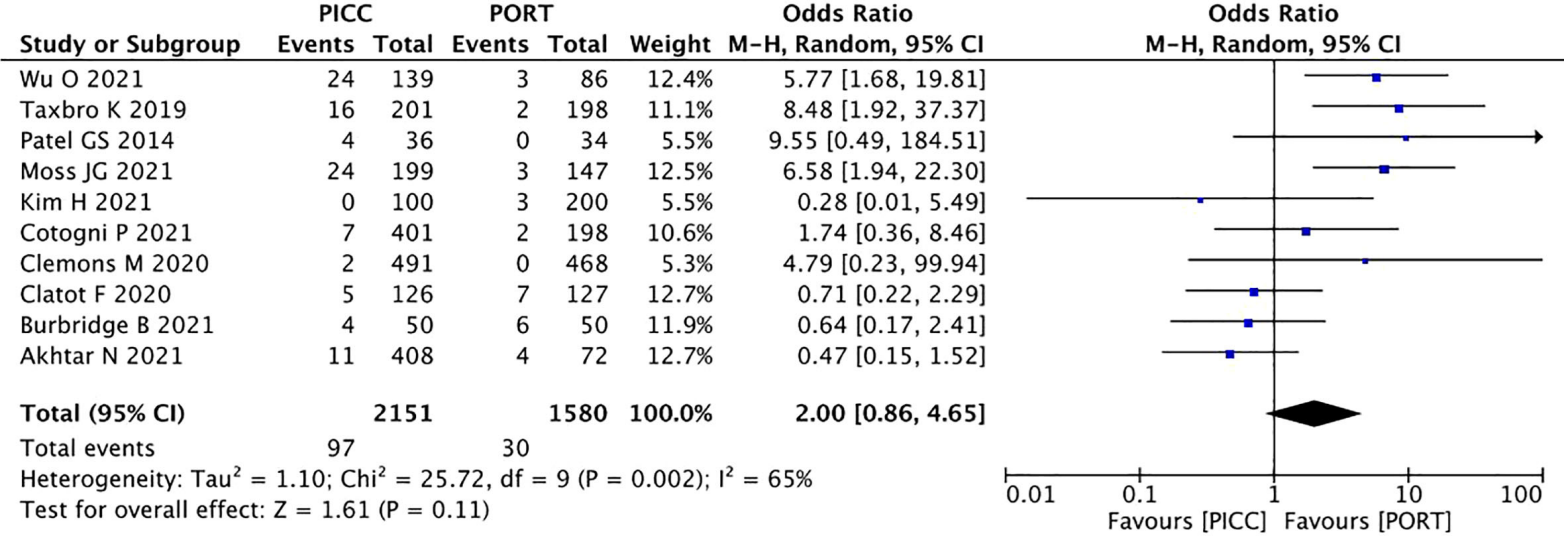
Pooled analysis of DVT comparing PICC with PORT.

A fixed-effects model was used to pool the thrombosis data because heterogeneity across the included studies was low. The pooled data showed the catheter-related thrombosis to be more frequent in patients with a PICC (OR=2.84, 95% CI=1.97–4.11, P<0.00001) (**Figure 4**), while the rate of the DVT did not reach a statistically significant level (OR=2.00, 95% CI=0.86–4.65, P=0.11) (**Figure 5**).

##### 3.2.2.2 Allergic reaction

**Figure 6:**
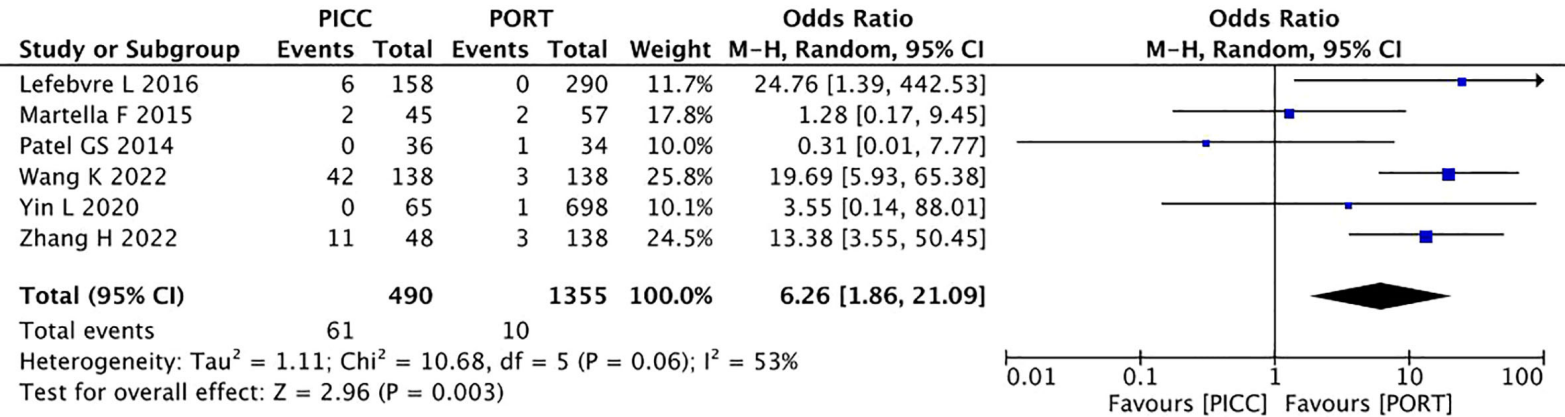
Pooled analysis of allergic reaction comparing PICC with PORT.

A significant heterogeneity was noted among the three studies (I^2^ =53%, P=0.06). The pooling allergic reaction data showed statistical significance in the PICC group (OR=6.26, 95% CI=1.86–21.09, P=0.003). Thus, the PICC regimen was associated with a higher rate of allergic reactions (**Figure 6**).

##### 3.2.2.3 Infection

**Figure 7:**
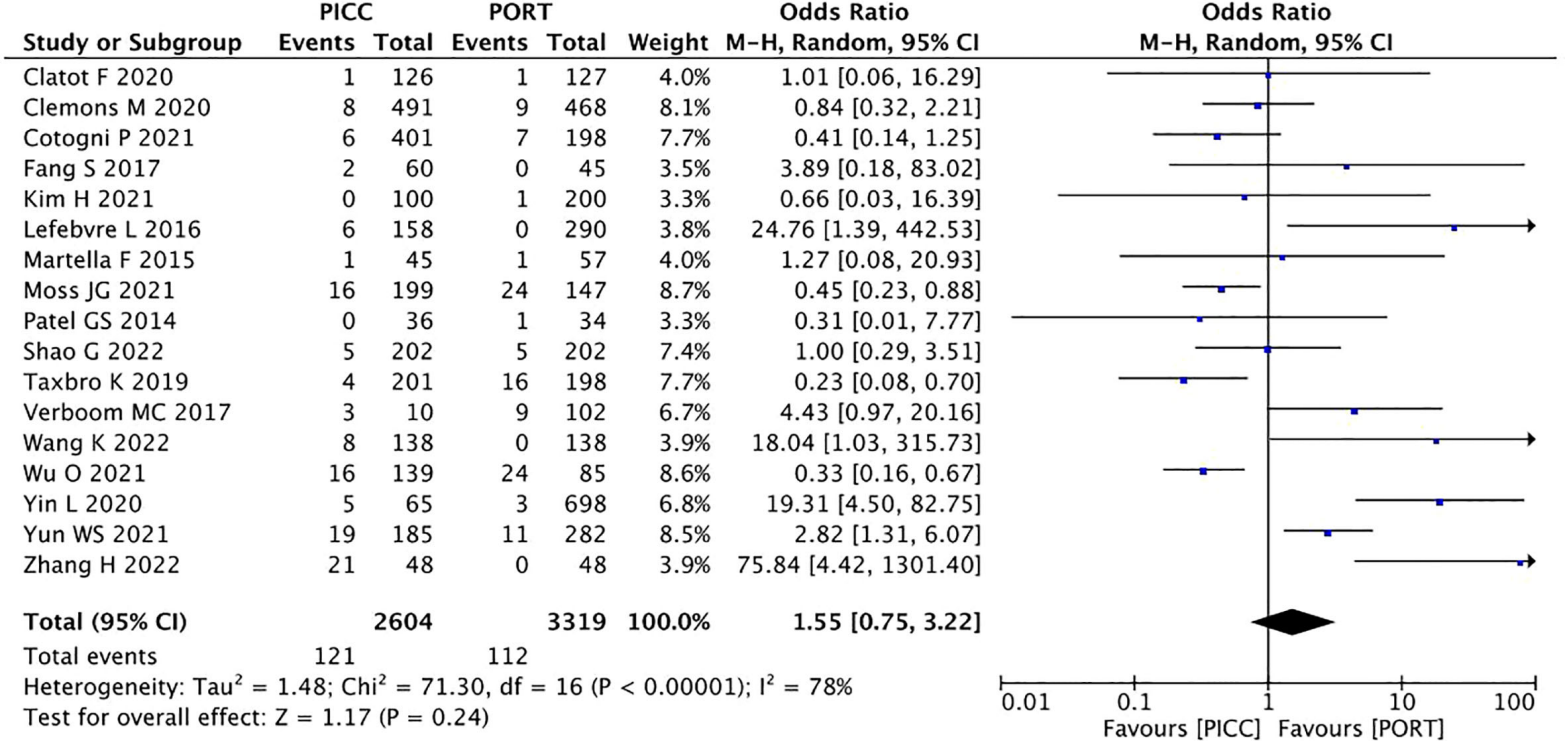
Pooled analysis of infection comparing PICC with PORT.

In terms of infection, a random-effects model was used, causing low heterogeneity across the included studies (I^2^ =78%, P<0.00001). The pooled result showed that PICC was non-inferior to the PORT group (OR=1.55, 95% CI=0.75–3.22, P=0.24) (**Figure 7**).

#### 3.2.3 Publication bias

**Figure 8:**
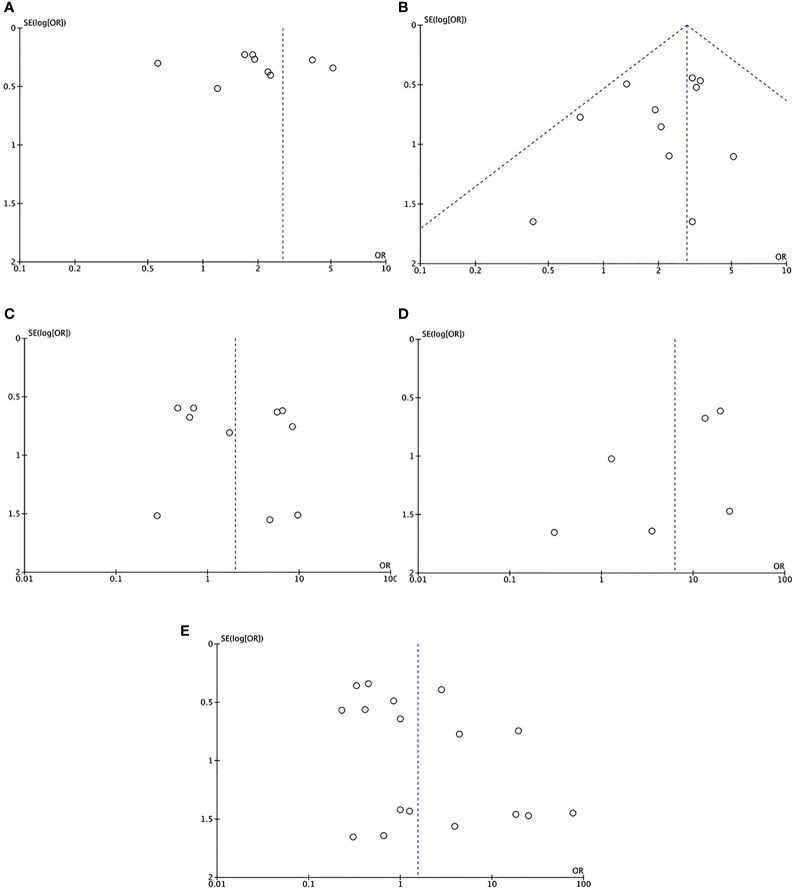
The funnel plots of the overall adverse effects **(A)**, catheter-related thrombosis **(B)**, deep vein thrombosis **(C)**, allergic reaction **(D)**, and infection **(E)**.

Forest plots were used to present publication bias. **Figure 8** shows funnel plots of the overall adverse effects (A), catheter-related thrombosis (B), deep vein thrombosis (C), allergic reaction (D), and infection (E).

## 4 Discussion

Despite an important management decision in clinical practice, little is known regarding the benefits and relative risks of the various methods of venous access in patients with cancer receiving chemotherapy. CVCs are associated with a high budgetary impact on cancer patients and place patients at risk of complications associated with catheters that could differ between PICCs and PORTs (19).

Today is an era that focuses on safety and raising cost awareness. Owing to the limited cost evidence, our meta-analysis only compared the complication rates of port catheters and PICC. The results indicated that PICCs were associated with a higher risk of catheter-related complications than PORTs, revealing that PICCs are an independent risk factor for device-related complications.

Consistent with previous studies (4, 5, 37), a higher rate of thrombosis and allergic reactions with PICC than with PORT was found in our study. Thrombosis was the most common complication. The pathogenesis of CR thrombosis is multifactorial and involves several risk factors (38, 39). Vascular injury, blood stasis, hypercoagulability related to cancer, and the catheter itself are considered classic contributors (Virchow’s triad) to venous thrombosis. Compared with PORT, PICCs are inserted into smaller-diameter veins and longer catheters. Therefore, this increased contact between the PICC and the vascular wall leads to vascular endothelium damage and blood flow reduction (17). However, the deep vein thrombosis did not reach statistical significance. Some elements, such as the study location and type of PICC-inserting provider, have been reported to be associated with the risk of vein thrombosis. A meta-analysis by Wang et al. (8) reported that PICC increased the risk of vein thrombosis compared with PORT in non-Asian countries, and no significant difference was observed in Asian countries. In addition, differences in the type of PICC-inserting provider and pooled results may affect the risk of vein thrombosis between the two groups. Considering the limitations of the included studies, limited data could not be obtained to explore the subtype elements affecting the interpretation of the results. Further investigation of vein thrombosis based on specific subtypes may help in making informed treatment decisions while maintaining a manageable safety profile.

With regard to the incidence of infection, the pooled results showed that PICC was non-inferior to PORT. To the best of our knowledge, this result may have been an underestimate. The extremities of a PICC remain outside the body and can increase the risk of bacterial infection on the outer edge of the catheter compared with implantable ports. Weekly maintenance inhibits cutaneous infectious side effects at the insertion site, whereas repeated handling and flushing may be associated with bacteremia (17). Some studies have shown that all nurses received relevant education in maintenance of PICC, and professional nurses and nurse education in aftercare led to a decrease in the rate of PICC complications in their clinical work (40).

Pool analysis uses a well-maintained and updated database. However, the retrospective nature of all the included studies may have influenced the comparison of catheter-related outcomes. Additionally, publication bias led to heterogeneity among the included studies. All studies in our meta-analysis used a wide range of malignancies and variations in specific methods of catheterization, catheter material, and catheter maintenance methods. Moreover, our study did not evaluate the cost efficiency between the two groups. Cost effectiveness should also be considered when adopting central access devices for patients with cancer.

## 5 Conclusion

This study found that patients with PICC had a higher rate of overall adverse events than those with PORT. The results provide practitioners with a better knowledge of adverse events between the two CVCs and will be of assistance when choosing an appropriate CVC for the administration of chemotherapy for cancer patients. When making clinical decisions, the safety of venous access and optimal patient satisfaction must be prioritized.

## Data availability statement

The original contributions presented in the study are included in the article/supplementary material. Further inquiries can be directed to the corresponding authors.

## Author contributions

YL and AW contributed to study design. LL and WL conducted the literature search, acquired the data and performed data analysis. LL wrote the article. All authors contributed to the article and approved the submitted version.
